# Lingual cyst with respiratory epithelium: The importance of differential diagnosis

**DOI:** 10.17305/bjbms.2020.4716

**Published:** 2021-06

**Authors:** Fabrizio Cialente, Giulia De Soccio, Vincenzo Savastano, Michele Grasso, Michele Dello Spedale Venti, Massimo Ralli, Mara Riminucci, Marco de Vincentiis, Alessandro Corsi, Antonio Minni

**Affiliations:** 1 Department of Sense Organs, University Sapienza of Rome, Rome, Italy; 2 UOSD Pediatric ENT, DAI Head-Neck, University Hospital Policlinico Umberto I, Rome, Italy; 3 Department of Molecular Medicine, University Sapienza of Rome, Rome, Italy; 4 Department of Oral and Maxillo-Facial Surgery, University Sapienza of Rome, Rome, Italy

**Keywords:** Lingual cyst with respiratory epithelium, differential diagnosis, tongue, lingual cysts

The lingual cyst with respiratory epithelium (LCRE) is a very rare congenital cyst of the tongue, floor of the mouth, pharynx, or hypopharynx with 21 cases reported in the literature [1,2].

Differential diagnosis is very important for patients presenting with lingual cysts, as this may impact treatment and follow-up. The LCRE should be included in the different diagnosis of a dermoid cyst [3], teratoid cyst [4], epidermoid cyst [5], thyroglossal duct cyst [6], lymphoepithelial cyst [7], and mucocele or ranula [8]. Each entity has a peculiar histologic presentation, although the clinical aspect may be very similar [1]. The dermoid cyst is lined by a keratinized squamous epithelium and contains skin appendages in the cyst. The epidermoid cyst is similar to the dermoid cyst but is characterized by non-keratinized squamous epithelium and has a lumen filled with keratin. The teratoid cyst contains derivatives of the endoderm, ectoderm, and/or mesoderm. The thyroglossal duct cyst is usually lined by columnar, stratified squamous epithelium, or an intermediate transition type of epithelium, with the mandatory presence of thyroid tissue in the cyst wall. The lymphoepithelial cyst is identified by the presence of lymphoid aggregates in the cyst wall. A mucous retention cyst, so-called mucocele or ranula, contains mucin and granulation tissue [1].

In order to differentiate the LCRE from other types of developmental cysts, Manor et al. [9] recommended the use of histologic descriptive terminology. According to that classification scheme, the epithelial lining of LCRE is composed predominantly by respiratory tract epithelium-pseudostratified ciliated cuboidal and columnar, differentiating it from the most commonly observed lingual alimentary cyst, mainly lined by gastric or intestinal mucosae. However, many reports in the literature described the epithelial lining of the lingual cyst as composed of both types of epithelium [9].

The pathogenesis of LCRE is unknown, but it most likely represents a congenital abnormality that arises from a misplacement of undifferentiated cells of the ventral portion of the foregut in week 4 of embryonic development [1,9]. In the 3^rd^ week of embryonic development, the foregut divides into a ventral part, containing components of the endoderm that lead to the development of the laryngo-tracheo-bronchopulmonary tree, and a dorsal part that becomes the proximal gastrointestinal tract. During this time of differentiation, embryonal rests may be misplaced and entrapped in the pharyngeal arches (which contains the developing tongue), due to their proximity with the primitive foregut. These entrapped rests, which are pluripotential, can differentiate into respiratory epithelium and form a lingual cyst [10].

We have recently treated a case of a 44-year-old male with a palpable, soft, tender mass occupying the entire width of the tongue, causing a mild restriction of tongue movement and elevation of the anterior floor of the mouth. Magnetic resonance imaging (MRI) showed a heterogeneously hyperintense cystic mass measuring 6 × 6 × 4 cm in size, located in the sublingual space (Figure 1). Histologic examination of the surgical specimen revealed a cystic lesion lined by well-differentiated ciliated, pseudostratified, columnar epithelium (Figure 2A and B). Immunohistochemical analysis, performed as described previously [10,11], revealed the respiratory-type origin of the epithelial cell lining. Indeed, the epithelial lining cells were immunoreactive for cytokeratin 7 (CK7) and thyroid transcription factor 1 but not for CK20 and thyroglobulin (Figure 2C-E). In addition, a thick smooth muscle desmin-positive layer (Figure 2A and F) was present underneath the epithelial lining. Based on these findings, the lesion was classified in the spectrum of the oral foregut duplication cysts. More specifically, the respiratory type of the epithelial lining and the site of the lesion were *per se* consistent with the diagnosis of LCRE [9].

**FIGURE 1:**
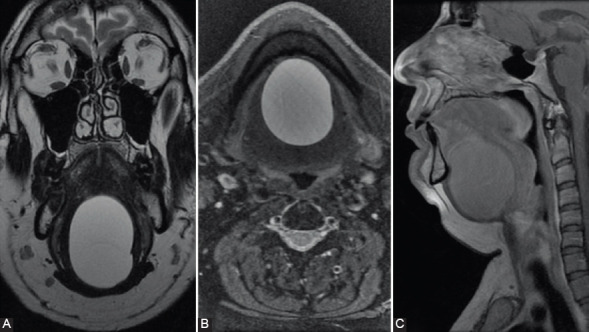
Magnetic resonance imaging of a patient with a lingual cyst with respiratory epithelium (LCRE) that demonstrates an approximately 6 cm cystic mass beneath the tongue in the coronal (A), axial (B), and sagittal planes (C). The lesion shows high signal on both basic (A) and fat-saturated T2-weighted (B) images, no contrast enhancement on T1 sequences (C). These aspects are in keeping with simple fluid collection. (A) COR T2 FSE; (B) AX FRFSE T2 Fat Sat; (C) SAG T1 FSE + contrast.

**FIGURE 2:**
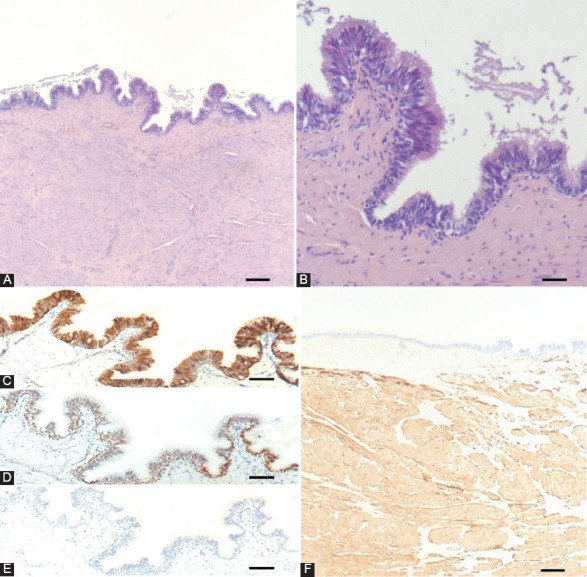
Lingual cyst with respiratory epithelium (LCRE): low-power magnification of the cyst wall is illustrated in (A). The epithelial layer consists of ciliated, pseudostratified, columnar cells (B) which are immunoreactive for cytokeratin 7 (C) and thyroid transcription factor 1 (D) but not for thyroglobulin (E). The thick smooth muscle cell layer underneath the epithelial lining (A) is highlighted by desmin immunostaining (F). A and B: hematoxylin and eosin. Bars: 200 mm in A and F; 100 mm in B; 80 mm in C, D, and E.

To date, 21 cases of LCRE have been reported in the literature [1]. Several case reports that were considered in the previous reviews as LCRE were excluded because not lined with the Manor’s histological criteria. According to that, only 7 (cases 4, 6, 7, 9, 11, 12, and 15) of the 16 cases reported by Wiersma et al. [12] and one case in the series of 16 reported by Chai et al. [4] were included in this review (Table S1). The age of presentation ranged from 6 months to 42 years of age, with a slight male predilection. Except for 5 adult cases, all cysts occurred in the pediatric age. Clinically, the lingual cyst appears on the dorsal tongue or the floor of the mouth; a common sign is the swelling of the tongue which causes difficulty in eating, drinking, speaking, and breathing. All patients were treated by complete excision of the cyst or the swelling marsupialization. No recurrence was reported [1].

In conclusion, various well-established types of developmental cysts have been described in the tongue. The LCRE represents a distinct entity histologically characterized by the presence of respiratory tract epithelium, pseudostratified ciliated columnar and cuboidal, with the absence of any other structures within the cyst wall. These characteristics should always be considered as, due to its rarity, the LCRE is often overlooked with consequences on the treatment and prognosis of affected patients.

## Figures and Tables

**TABLE S1:**
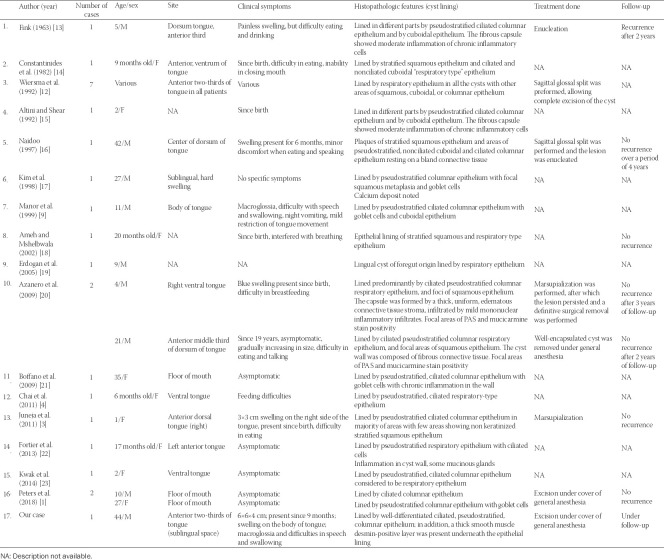
Cases of lingual cyst with respiratory epithelium reported in the literature
